# Assessing recognition memory using confidence ratings and response times

**DOI:** 10.1098/rsos.150670

**Published:** 2016-04-13

**Authors:** Christoph T. Weidemann, Michael J. Kahana

**Affiliations:** 1 Department of Psycholoy, Swansea University, Singleton Park, Swansea SA2 8PP, UK; 2 Department of Psychology, University of Pennsylvania, 3401 Walnut Street, Philadelphia, PA 19104, USA

**Keywords:** recognition memory, response times, confidence ratings, receiver operating characteristic

## Abstract

Classification of stimuli into categories (such as ‘old’ and ‘new’ in tests of recognition memory or ‘present’ versus ‘absent’ in signal detection tasks) requires the mapping of internal signals to discrete responses. Introspective judgements about a given choice response are regularly employed in research, legal and clinical settings in an effort to measure the signal that is thought to be the basis of the classification decision. Correlations between introspective judgements and task performance suggest that such ratings often do convey information about internal states that are relevant for a given task, but well-known limitations of introspection call the fidelity of this information into question. We investigated to what extent response times can reveal information usually assessed with explicit confidence ratings. We quantitatively compared response times to confidence ratings in their ability to qualify recognition memory decisions and found convergent results suggesting that much of the information from confidence ratings can be obtained from response times.

## 1. Introduction

**Figure 1.**
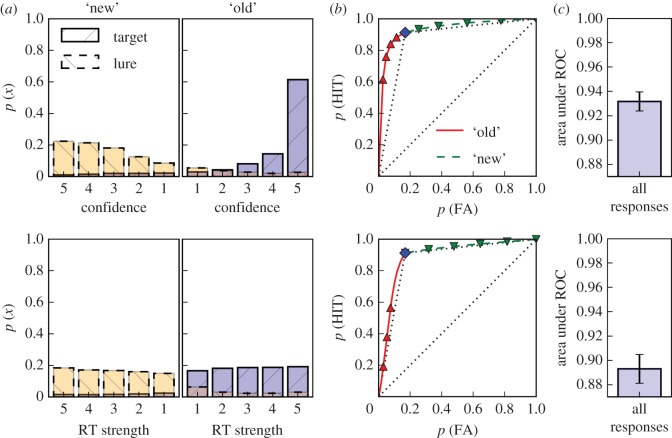
Response probabilities for bins of each measure (*a*) used to compute the ROC functions (*b*) with corresponding areas (*c*). Rows show analyses for confidence ratings and response times, respectively. For illustrative purposes, RT strength is shown partitioned into the same number of levels as there are confidence ratings (using equally-spaced quantiles) and the points on the ROC functions corresponding to these bins are indicated. However, smooth ROC functions taking advantage of the full resolution of the data are drawn and form the basis of the area calculations. The left-most points on the ROC functions correspond to the right-most bars in the left panels and subsequent points are calculated by cumulatively adding the probabilities for targets and lures to the hit and FA values, respectively. Probabilities for target and lures are shown with overlapping bar graphs with hatching as indicated in the legend (additional shading, blue for targets and yellow for lures, is added to help with the discrimination). The classification point (i.e. the point separating ‘old’ from ‘new’ responses) is shown as a diamond (solid-red and dashed-green parts of the ROC functions indicate the parts corresponding to ‘old’ and ‘new’ responses, respectively). Main diagonals as well as random ROC functions are shown as dotted lines in the ROC plots. The lowest value on the ordinate for the bar graphs on the right (0.87) corresponds to the area under the random ROC. Error bars on the area measure show the 95% confidence intervals.

Any assessment of recognition memory (and performance in other classification tasks) needs to separate the ability to distinguish the different stimulus classes (e.g. old and new items) from preferences for the different response classes (i.e. response biases). A large proportion of correct classifications of previously studied items as ‘old’ could reflect substantial ability to distinguish between old and new items or a tendency to liberally respond ‘old’ when presented with a recognition memory test. Signal detection theory (SDT) is a common analysis framework for tasks with two response classes [1]. Within this framework, the strength of the (internal) signal on which the classification is based is assumed to vary continuously and a criterion is placed to map the continuous signal onto a binary classification response. Responses can be classified as ‘hit’ (correct response in favour of a standard response class, e.g. ‘old’ in recognition memory tasks or ‘present’ in signal detection tasks) or ‘false alarm’ (FA; incorrect response in favour of a standard response class), as well as ‘correct rejection’ or ‘miss’ (correct and incorrect responses to the non-standard response class, respectively). With rising endorsement of the standard response class (when data from different response criteria are obtained), the way the cumulative hit rates increase relative to the cumulative FA rates indexes a classifier’s performance (a receiver operating characteristic (ROC) function; figure 1). To the extent that a classifier correctly distinguishes between the two stimulus classes, hit rates should initially rise faster leading to a concave ROC function.

Several indices of discriminability and response bias have been developed within the framework of SDT. Most of these indices are strongly dependent on the specific assumptions associated with a particular application of SDT. The area under an ROC function (AUC), however, provides an index of discriminability which does not depend on strong (and typically untested) assumptions about the distribution of internal states—it is simply a measure of ordinal separation of the two distributions indexing the responses to targets and lures, respectively. In contemporary research on perception and memory, ROC functions are almost exclusively constructed through putative manipulation of response criteria (e.g. by varying instructions, stimulus base-rates, or pay-off contingencies) or from introspective judgements (e.g. through confidence ratings with each level assumed to reflect a different response criterion). Because the former approach is particularly costly (requiring dedicated experimental blocks/sessions for each point on the ROC function), the use of confidence judgements is the de facto standard for the generation of ROC functions in recognition memory tasks.

Introspective judgements are perhaps the oldest empirical tool for psychological inquiry—they are regularly employed in research, legal and clinical settings [2,3], but have also long been recognized as problematic [4–9]. Correlations between introspective judgements and task performance suggest that such ratings often do convey information about internal states that are relevant for a given task [10–13], but similar correspondences are often observed between task performance and response latency [14]. Indeed response times play a central role in models accounting for performance in recognition memory (and other) tasks with sequential sampling or diffusion processes [15].

Research on the nature of confidence judgements has shown that they largely reflect ‘fluency’ of the response (i.e. the ease and speed with which it is generated), even when it is a poor index of performance [16–20]. In the light of the well-known relationship between such ratings and response fluency (often operationalized by response latency), the substantial effort required to solicit these ratings (which often take longer to execute than the response to which they apply), and the strong correspondence between response times and performance [14], it is remarkable that attempts to quantify discriminability through analyses of response time data are mostly limited to the fitting of sequential sampling models (which usually require large numbers of trials to estimate various parameters in addition to those directly reflecting the ability to distinguish old from new items).

Here, we raise the question to what extent we can quantify the discriminability of memory states independent from response biases on the basis of response latencies. To foreshadow the results, we show that despite absolute differences in the magnitude of performance indices derived from confidence ratings and response latencies, the relative pattern of these indices across various partitions of the data is remarkably similar.

### 1.1. Generating receiver operating characteristic functions from response times

Historically, a wide variety of dependent variables, including response times [11,21–38], latency of heart rate increase [27], response frequency [39], and firing rates of individual neurons [32,40], have been used for ROC construction. To construct an ROC function from a dependent variable, one has to assume or establish a mapping between this measure and the evidence for the classification response. A common way to construct ROC functions is to partition the dependent variable (e.g. response time) by the classification response and to sort it within these partitions on the basis of this mapping. For the case of response time, the usual assumption is that fast responses are based on a stronger signal/made with higher confidence than slow responses, such that, in the case of recognition memory, the inferred evidence that an item has been studied is weakest for fast ‘new’ responses, strongest for fast ‘old’ responses and intermediate for slow responses [22,24,41,42]. We refer to response times (RTs) ordered in this way as ‘RT strength’. This assumed relationship between signal strength and response time is well supported by findings that responses that are made with high confidence also tend to be made faster than those with low confidence [43,44]. We address occasional exceptions to this relationship in the Discussion section.^1^

### 1.2. Classification performance and signal strength

ROC functions constructed in this way are constrained to pass through the classification point (the point separating, say, ‘old’ from ‘new’ responses in tests of recognition memory or ‘signal present’ from ‘signal absent’ responses in signal detection tasks) as well as through the points where both hits and FAs are either zero or one. Consider, for example, a random measure that bears no relationship to the classification response such as the throw of a die. Just as with confidence ratings, one could interpret the outcomes of a die thrown after every classification response in a test of recognition memory such that the inferred memory strength of an item is weakest for a ‘new’ response with an outcome of six, strongest for an ‘old’ response with an outcome of six, and intermediate for lower casts. In the limit, the partitions of this variable within a given classification response contain equal proportions of correct and incorrect responses such that the rate of increase of hits and FAs in the ROC function is constant within each classification response. Thus, a ‘random ROC’ that reveals no information beyond that contained in the classification response is bilinear, connecting the origin to the classification point and that point to (1,1) with straight lines (shown together with the main diagonal as dotted lines in figure 1; [8,24,45]). The AUC therefore conflates classification performance with the measure’s ability to reflect the signal underlying the classification decision. A relative index of how much information a particular measure contains about the signal underlying the classification response can be obtained by subtracting the area under the random ROC from that under the ROC of interest [24]. Previous applications of this method have shown that response times and other measures contain significant information that qualifies a classification response at levels that sometimes approached, but never exceeded that in confidence ratings [24,33,38].

In order to assess to what extent response times can reveal information similar to that obtained by confidence ratings, we administered a recognition memory test that first asked for a binary recognition decision followed by a confidence rating. This set-up allowed us to directly compare the time taken for the recognition decision with the subsequent introspective judgements.

## 2. Material and methods

### 2.1. Participants

We obtained data from the Penn Electrophysiology of Encoding and Retrieval Study which asked participants to contribute data for a total of 20 sessions each. From all participants we selected young adults (ages 18–30 years) who provided data from at least seven sessions. We excluded trials with invalid confidence responses and those with response times for binary old–new judgements below 300 ms or above 3000 ms (a total of 3% of the full dataset). From the remaining data, we eliminated 121 sessions (about 3% of the data) that did not contain at least one ‘old’ and one ‘new’ response for both targets and lures. Some analyses partitioned the targets into those that were previously recalled and those that were not (see below for details). For those analyses, we additionally required that sessions contained at least one ‘old’ and one ‘new’ response for both types of targets which excluded a further 540 sessions (16% of the remaining dataset). These exclusion criteria yielded a total of 171 participants (of which only 10 provided data from fewer than 10 sessions for the general analyses, with 24 participants providing data from fewer than 10 sessions for the analyses partitioning the data by previous recall). The total number of analysed sessions was 3120 for the general analyses and 2580 for the analyses partitioning the data by previous recall.

### 2.2. Experimental task

Each session included multiple pairs of study lists followed by recall tasks. Details of study conditions and recall tasks varied across sessions (see [46] for details), but in all cases participants studied words presented on a computer screen before being probed to recall the previously presented words. The current study focuses mostly on a final recognition test at the end of each session. Participants were shown one word at a time and asked to indicate whether each word was presented in any of the previous study lists that had been shown in this session. Participants answered verbally by speaking either ‘pess’ or ‘po’ into a microphone to answer in the affirmative or negative, respectively (‘yes’ and ‘no’ were replaced by ‘pess’ and ‘po’ to equalize the initial phoneme in an effort to allow more precise measurements of response latencies; response time was only measured in relation to the initial recognition memory decision and not with respect to the confidence rating). Following the ‘old’/‘new’ classification, participants were asked to rate their confidence in their classification response on a 5 point scale from ‘very unsure’ to ‘very sure’ by either speaking a number between 1 and 5 into the microphone (most participants) or by pressing the corresponding number key on the keyboard. The proportion of lures in the recognition memory test was varied across sessions, but this manipulation had miniscule effects on performance and was not a focus of the current investigation. Participants indicated that they had finished speaking by pressing the space key on a computer keyboard both after the classification response and after the confidence rating (response times were only measured as the latency of the verbal classification response). Immediately after participants indicated that they had finished the confidence rating they received brief (randomly jittered between 100 and 200 ms) visual (‘Correct’ in green or ‘Incorrect’ in red) and auditory feedback on their classification decision (feedback was automatically generated with custom speech recognition software). After offset of the feedback, the screen turned blank for a variable interval uniformly distributed between 800 ms and 1200 ms before the next test word was presented. All stimulus presentation and recording of voice and button-press responses were done with PyEPL [47]. Some analyses condition recognition memory performance on whether or not a given target item was recalled in any of the recall periods of that session, but we make no other reference to performance in the recall periods of this experiment. Electroencephalography recordings were obtained but are not further discussed in this article.

## 3. Results

Means of the distribution of all response times ranged from 773 ms for hits (s.d.=321) to 1065 ms for misses (s.d.=459), with response times for false alarms (*M*=943, s.d.=502) and correct rejections (*M*=967, s.d.=389) falling between those two extremes. Visual inspection of the response time distributions confirmed that their shapes were typical for response time distributions.

### 3.1. Assessing memory strength with C- and L-ROC functions

Figure 1 illustrates the construction of the ROC functions based on confidence ratings (C-ROC; top row) and response latency (L-ROC; bottom row). The figure breaks down the steps of constructing and assessing ROC functions to illustrate how this process generalizes across dependent variables. The common case of using confidence ratings to construct ROC functions is illustrated in the top row; the bottom row mirrors these steps for response latencies. We followed this procedure separately for each experimental session, averaged the sessions for each participant and show the mean across participants in figure 1. It is important to note that as long as some relationship between a dependent variable and the strength of the signal underlying the classification response can be assumed or established, the same procedure can be used to evaluate to what extent this variable is able to qualify the classification decision. Similarly, this procedure is completely agnostic with respect to the nature of the classification decision and, indeed, previous applications of this procedure have almost exclusively focused on signal detection/perceptual discrimination tasks [24]. A particular feature of confidence ratings is that they are usually discrete, whereas many other variables that could be used to qualify the signal underlying classification decisions (such as response times and physiological recordings) are continuous. To better illustrate the correspondence with confidence ratings, the bottom panel of figure 1*a* shows RT strength binned into the same number of bins as there are in our confidence scale (using equally spaced quantiles). All analyses, however, are based on the raw latency data which is why the L-ROC function does not connect the indicated points with straight lines. The curvature reflecting the use of the full resolution of the latency data is difficult to discern, but most prominent for the lowest strength ‘old’ responses.

Though uncommon (and impractical for continuous variables), the response probability plots in figure 1*a* contain the same information as the corresponding ROC functions, albeit at a lower resolution for our latency data for illustrative purposes as explained above. Cumulatively adding the response probabilities (starting with the strongest ‘old’ responses) for targets and lures to the hit and FA probabilities, respectively, traces out the ROC function (figure 1*b*). The response probability plots indicate that for both confidence ratings and RT strength, stronger responses tended to be associated with higher response probabilities for correct responses and lower response probabilities for incorrect responses—a trend that is more easily quantified on the basis of the resulting ROC functions. As is evident from figure 1*c*, the L-ROC is closer to the random ROC than the C-ROC, resulting in a significant difference between the areas under the C-ROC and the L-ROC functions (*t*_170_=13.609, s.e.=0.003, *d*=1.041, *p*<0.001). As is also clear from the figure, the areas under both ROC functions exceeded those under the random ROC functions, effects which turned out to be substantially larger than the difference between the two areas (*t*_170_=109.312, s.e.=0.004, *d*=8.359, *p*<0.001 and *t*_170_=65.243, s.e.=0.006, *d*=4.989, *p*<0.001, for C-ROCs and L-ROCs, respectively).

**Figure 2.**
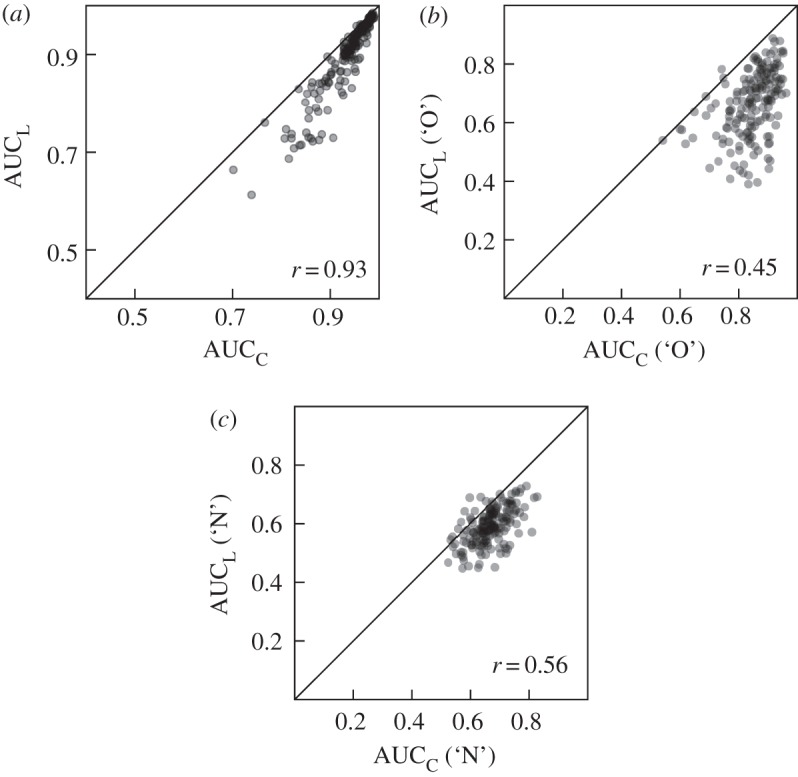
Scatter-plots comparing areas under the ROC curve (AUC) for confidence ratings (C) and response latency (L). Corresponding correlations and the main diagonal are indicated in each panel. Data points correspond to the average AUCs across all sessions for each participant. Individual data points are transparent such that darkness indicates density of points in a given area. (*a*) Comparison of the areas under the entire ROC functions. (*b*) Comparison of the areas under the ROC functions for ‘old’ (O) responses only. (*c*) Comparison of the areas under the ROC functions for ‘new’ (N) responses only. Note that the scales in (*a*) differ from those in the other two panels.

To investigate the correspondence between the AUC for C-ROC and L-ROC functions (AUC_C_ and AUC_L_, respectively), we analysed the correlation of these and related measures. Figure 2*a* shows a scatter-plot of AUC_C_ and AUC_L_ that reveals a very close correspondence between both areas (*r*=0.93, *t*_169_=44.714, *p*<0.001). This correlation, however, is inflated by the fact that both ROC functions are constrained to pass through the classification point. To illustrate this issue, it may help to point out that both AUC_C_ and AUC_L_ are also expected to strongly correlate with the AUC for the random ROC function. We interpret each dependent measure as reflecting evidence for the particular classification response and the hit and false alarm rates associated with that classification define a point on any corresponding ROC function. Thus, AUCs will be larger for ROC functions reflecting higher classification performance regardless of what measure is used to construct the ROC function (and indeed even if that measure contains no information about the classification decision at all). Confidence ratings can also be used to solicit an absolute measure of evidence for one and against the other alternative. Used this way, extreme (low or high) ratings indicate high confidence for one or the other stimulus class, respectively, with intermediate ratings indicating low confidence. For some other measures, such as choice RT, it does not seem possible to extract the direction of the choice along with its confidence without a separate classification decision. When each end of the rating scale indicates support for one of the stimulus classes, a classification threshold (e.g. the middle rating option) is not always explicitly indicated, making it difficult to identify a classification point on the corresponding ROC function. However, as long as a classification is taking place, the fact remains that classification performance and the dependent measure’s ability to reflect the signal underlying the classification decision jointly determine the shape of (and hence the area under) the corresponding ROC function.

### 3.2. Classification-response specific receiver operating characteristic functions

**Figure 3.**
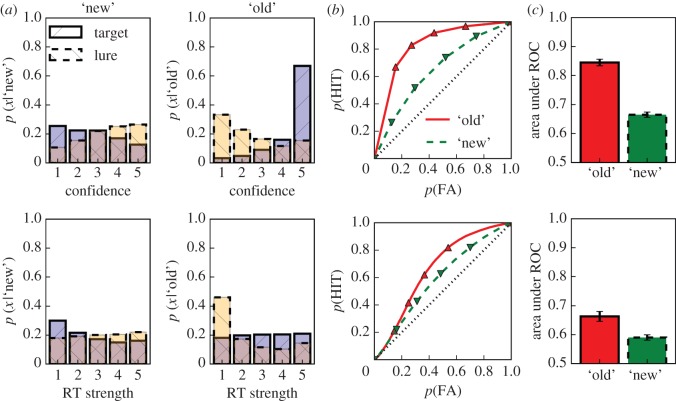
Conditional response probabilities for bins of each measure (*a*) used to compute conditional ROC functions (*b*) with corresponding areas (*c*). Same data as in figure 1, but probabilities are conditioned on the respective classification responses. Separate ROC functions for ‘old’ and ‘new’ judgements are generated by cumulatively adding target and lure probabilities with decreasing strengths to the hit and FA values, respectively, for ‘old’ responses and vice versa for ‘new’ responses. The corresponding AUCs with 95% confidence intervals are shown in (*c*). As in figure 1 blue and yellow shadings correspond to data from targets and lures, respectively, and red and green shadings correspond to data from ‘old’ and ‘new’ responses, respectively.

Another way to compare confidence ratings and response times as measures of memory strength, without contamination from classification performance, is by assessing their ability to qualify classification responses separately for ‘old’ and ‘new’ judgements. Figure 3 illustrates how the data for ‘old’ and ‘new’ judgements can be separately used as the bases for the calculation of classification-alternative specific ROC functions. Figure 3 shows the same data as figure 1, but this time response probabilities are conditioned on the classification response. As in figure 1, RT strength is binned in figure 3*a* and the points corresponding to these bins are indicated on the respective ROC functions in figure 3*b*. This binning again serves to illustrate the correspondence between the approaches for confidence ratings and response latencies, and we used the full resolution of the response time data in the generation of ROC functions and for corresponding analyses (the curvature of the lines connecting points on the L-ROC functions which reflects our use of the raw latency data is clearly discernible in figure 3*b*). Whereas the response probabilities for targets and lures across both classification responses each add up to 1.0 in figure 1*a*, they add up to 1.0 within each classification response in figure 3*a*. The distribution of target and lure response probabilities across the response categories in figure 1*a* reflects the overall classification performance. By conditioning on the classification response in figure 3*a*, classification performance does not affect the resulting ROC functions. We present this approach as a novel way to analyse measures qualifying classification responses that allows for the separate assessment of such measures for each response class. We comment further on this approach in the Discussion section and we illustrate the calculation of different ROC functions with a worked-out example in appendix A.

Given that figure 3 is based on the same data as figure 1, it is not surprising that it, too, indicates that stronger responses are associated with higher probabilities for correct responses and lower probabilities for incorrect responses—a trend that is more easily discernible in figure 3*a* for both response alternatives owing to the conditioning. These response probabilities contain the same information as the corresponding ROC functions (albeit at a lower resolution for L-ROCs owing to the binning as explained above). They can be used to generate separate ROC functions for both response alternatives (‘old’ and ‘new’ in this case) by cumulatively adding response probabilities for targets and lures with decreasing strengths to the hit and FA probabilities, respectively, for the standard response class (‘old’ in this case) and vice versa for the other response alternative.

The areas under these ROC functions indicate to what extent the respective dependent variable (confidence ratings or RT in our case) qualifies a given classification response alternative (‘old’ or ‘new’ in our case). Areas under all ROC functions in figure 3 clearly exceeded 0.5 (*t*_170_=18.933–59.921, s.e.=0.005–0.009, *d*=1.448–4.582, all *ps*<0.001) confirming that both measures contain additional information about each classification response alternative. A large difference between the areas under the ROC functions for ‘old’ and ‘new’ responses is also apparent (*t*_170_=36.076, s.e.=0.005, *d*=2.759 and *t*_170_=11.337, s.e.=0.006, *d*=0.867, for confidence ratings and response latencies, respectively; both *ps*<0.001). The differences between the areas under the corresponding C- and L-ROC functions are also substantial (*t*_170_=23.038, s.e.=0.008, *d*=1.762, and *t*_170_=17.781, s.e.=0.004, *d*=1.360 for ‘old’ and ‘new’ responses, respectively; both *ps*<0.001). Figure 2*b*,*c* shows the relationship between AUC_C_ and AUC_L_ for ‘old’ and ‘new’ responses, respectively. These scatter-plots illustrate large correlations between AUCs derived from confidence ratings and RTs for both ‘old’ (*r*=0.45, *t*_169_=7.860, *p*<0.001) and ‘new’ (*r*=0.56, *t*_169_=11.053, *p*<0.001) responses that are not affected by the constraint of the full ROC functions to pass through the classification point.

**Figure 4.**
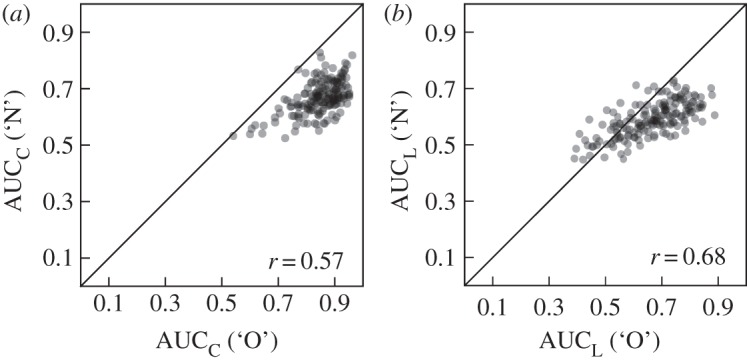
Scatter-plots comparing areas under the ‘old’ (‘O’) and ‘new’ (‘N’) ROC functions (AUC) for confidence ratings (C; (*a*)) and response latency (L; (*b*)). Corresponding correlations and the main diagonal are indicated in each panel. Individual data points are transparent such that darkness indicates density of points in a given area.

As illustrated in figure 3, both confidence ratings and response latencies are much better able to qualify ‘old’ responses than ‘new’ responses. Nevertheless, it seems reasonable to ask to what extent these measures are correlated across classification responses (e.g. do large areas under the C-ROC (L-ROC) for ‘old’ responses coincide with large areas under the C-ROC (L-ROC) for ‘new’ responses?). Figure 4 illustrates substantial and similar relationships between AUCs for ‘old’ and ‘new’ responses for confidence ratings (*r*=0.57, *t*_169_=11.174, *p*<0.001) and response latencies (*r*=0.68, *t*_169_=15.548, *p*<0.001).

### 3.3. Previous recall as a proxy for memory strength

**Figure 5.**
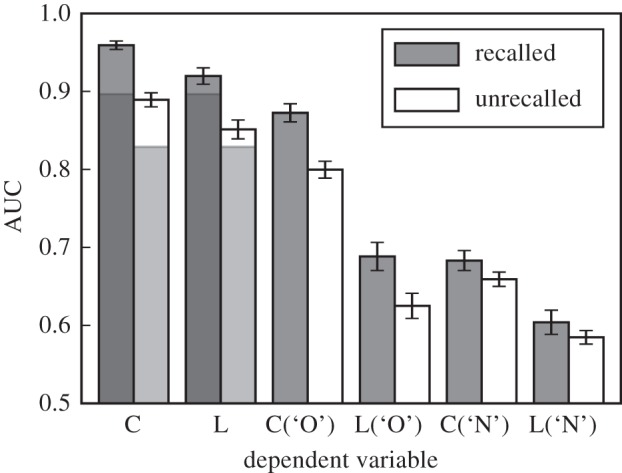
Mean areas under the ROC functions (AUC) across participants for targets that were previously recalled and those that were previously unrecalled within each session (the same set of lures were used in the calculation of both sets of AUCs). Separate AUCs are shown for ROC functions based on confidence ratings (C), response latency (L) as well as corresponding ROC functions conditioned on the classification response (‘O’: ‘old’, ‘N’: ’new’). The darker shadings in the lower parts of the C and L bars indicate the portions of the total area that are attributable to the area of the random ROC. Error bars show the 95% confidence intervals.

When comparing indices of sensitivity that are based on different dependent measures, it is useful to assess how these measures fare when sensitivity varies. Comparisons of performance for conditions with different levels of memory strength have also been used in tests aiming to distinguish threshold accounts of recognition memory from signal detection accounts [48]. It is therefore important to assess to what extent estimates of sensitivity based on confidence ratings and response times covary across various levels of memory strength. A close correspondence across dependent measures would suggest that related findings based on confidence ratings may generalize to response times—a finding that would need to be confirmed by detailed modelling. Even though we did not manipulate sensitivity directly, the set-up of the current experiment allowed us to compare performance for groups of targets that can reasonably be expected to differ in sensitivity. Specifically, we compared performance for targets that have been previously recalled to that for items that have not been recalled in any of the recall periods that preceded the recognition memory test. Across all included sessions, the mean proportion of previously recalled targets was 61% (s.d.=16%). Figure 5 shows the mean AUCs based on previously recalled versus unrecalled targets for confidence ratings, response latencies and the corresponding AUCs for ROC functions that are conditioned on the classification response (cf. figure 3). It is clear from the figure that recognition memory was lower for targets that were not previously recalled (as indicated by the shorter shaded areas outlining the areas for the random ROCs that are only based on classification performance; *t*_170_=36.265, s.e.=0.002, *d*=2.773, *p*<0.001). For both sets of items, confidence ratings and response latencies effectively qualified classification decisions as indicated by AUCs for classification response-specific ROC functions exceeding 0.5 (*t*_170_=13.263–63.322, s.e.=0.004−0.009, *d*=1.014–4.842, *ps*<0.001). The difference between the AUCs corresponding to the C-ROC as well as the L-ROC and the random ROC appears to be similar for AUCs based on recalled versus unrecalled trials (0.063 versus 0.060 and 0.023 versus 0.022 for AUCs based on C-ROCs and L-ROCs, respectively). However, this similarity is deceiving, because these differences have different baselines anchored on the random AUCs for recalled (0.897) and unrecalled (0.829) targets. The AUCs based on the ROC functions that are conditioned on the classification response (the four right-most sets of bars in figure 5) reveal that both confidence ratings and response latencies were somewhat more effective at qualifying recognition decisions for previously recalled items, especially when these were classified as ‘old’. A 2 (recalled status) ×2 (response) ×2 (dependent measure) repeated measures ANOVA on the AUCs that are conditioned on the classification response (i.e. the four right-most sets of bars in figure 5), confirmed large main effects (*F*_1,170_=332–645, MSE=10.17–91.08, $\eta_{p}^{2}=0.66$–0.79, *ps*<0.001) as well as large interactions between recalled status and response (*F*_1,170_=130, MSE=3.39, $\eta_{p}^{2}=0.43$, *p*<0.001) and between response and dependent measure (*F*_1,170_=310, MSE=13.85, $\eta_{p}^{2}=0.65$, *p*<0.001).^2^

**Figure 6.**
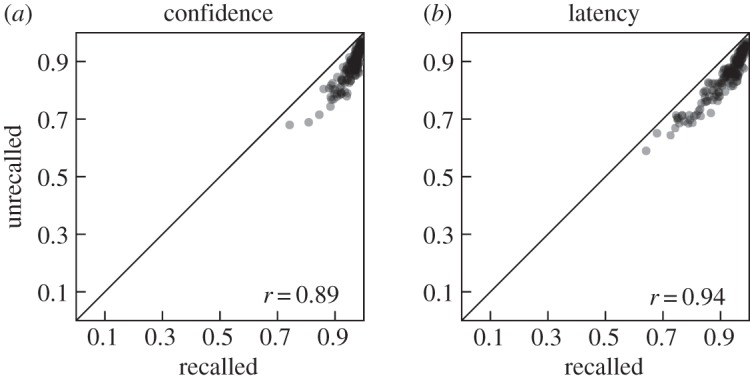
Scatter-plots comparing areas under the ROC functions for targets that were previously recalled and those that were previously unrecalled within each session (the same set of lures were used in the generation of both ROC functions). Separate scatter-plots are shown for ROC functions based on confidence ratings (*a*) versus response latency (*b*). Corresponding correlations and the main diagonal are indicated in each panel. Individual data points are transparent such that darkness indicates density of points in a given area.

**Figure 7.**
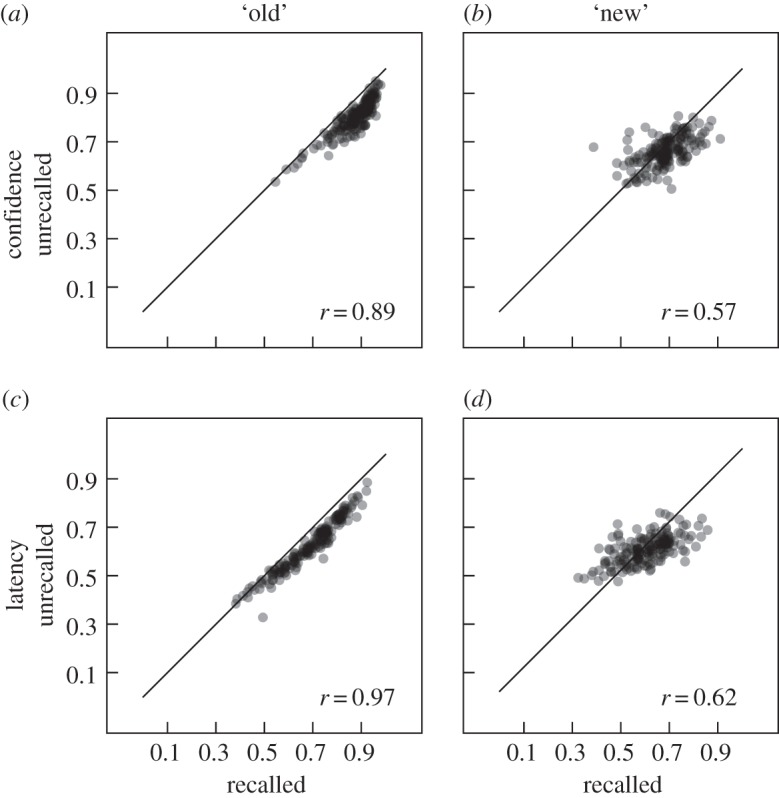
Scatter-plots comparing areas under the ROC functions for targets that were previously recalled and those that were previously unrecalled within each session (the same set of lures were used in the generation of both ROC functions). Separate scatter-plots are shown for ROC functions based on confidence ratings (top row, (*a*,*b*)) versus response latency (bottom row, (*c*,*d*)) and ‘old’ (left column, (*a*,*c*)) versus ‘new’ (right column, (*b*,*d*)) responses. Corresponding correlations and the main diagonal are indicated in each panel. Individual data points are transparent such that darkness indicates density of points in a given area.

**Figure 8.**
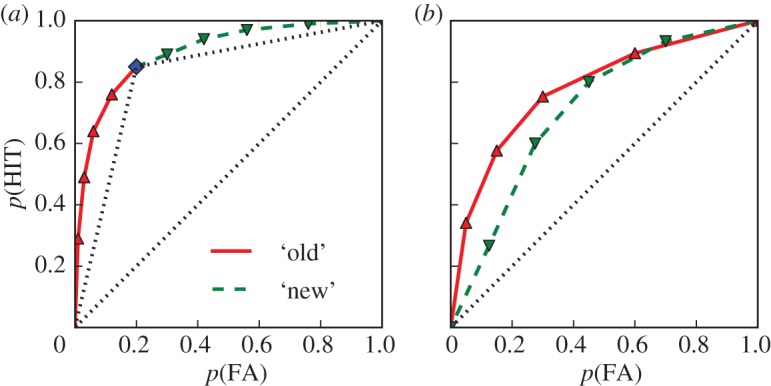
ROC functions based on fictional data shown in table 1. (*a*) An ROC function based on both ‘old’ and ‘new’ responses (cf. table 2). The classification point (i.e. the point separating ‘old’ from ‘new’ responses) is shown as a diamond (solid-red and dashed-green parts of the ROC functions indicate the parts corresponding to ‘old’ and ‘new’ responses, respectively). (*b*) Separate ROC functions for ‘old’ and ‘new’ judgements (cf. tables 3 and 4). Main diagonals as well as the random ROC function (*a*) are shown as dotted lines.

Despite this discrepancy between the effectiveness of C- and L-ROC functions for previously recalled and unrecalled targets, we would expect that in sessions where a given measure is effective at qualifying classification decisions involving previously recalled targets, it should also be effective at qualifying classification decisions involving previously unrecalled targets (and likewise for sessions where a given measure is less effective). Figure 6 shows the correlations between the AUCs based on previously recalled and unrecalled targets for C- and L-ROC functions. Both correlations are substantial and similar (*r*=0.89, *t*_169_=34.076 and *r*=0.94, *t*_169_=50.348, respectively, both *ps*<0.001). The corresponding correlations for AUCs based on ROC functions that are conditioned on the classification response are shown in figure 7 and also show substantial and similar correlations (*r*=0.89, *t*_169_=35.412, and *r*=0.97, *t*_169_=72.265 for ‘old’ responses as well as *r*=0.57, *t*_169_=11.158 and *r*=0.62, *t*_169_=12.989 for ‘new’ responses, all *ps*<0.001).

## 4. Discussion

Much of what we know about human behaviour comes from experimental work asking participants to judge a stimulus as belonging to one of two classes. Examples include tasks asking participants to distinguish studied from unstudied items (e.g. to investigate recognition memory—our focus in this paper), tasks asking participants whether two stimuli are identical or not (e.g. to establish a detection threshold), tasks asking which of two stimuli is larger/better on some dimension (e.g. to study inference and/or preference) and tasks asking participants to match stimuli to categorical labels (e.g. to study knowledge and/or perception). Most theories of cognitive processes that are measured with these tasks, assume that a continuous signal (e.g. strength of a memory, percept or preference) is somehow thresholded to produce a binary response (e.g. ‘old’ versus ‘new’ in a recognition memory task or ‘signal present’ versus ‘signal absent’ in a signal detection task). Attempts to characterize this signal, however, have mainly focused on aggregating large numbers of trials (e.g. to compare average levels of evidence across different experimental conditions) or on introspective judgements (e.g. by asking participants to rate their confidence in each classification decision). The latter measures have the distinct advantage of providing trial-by-trial assessments of the evidence underlying the classification decisions, but previous research suggests that they reflect an inference about this evidence (based on response fluency) rather than direct introspective access to it [16–20,49,50]. Furthermore, a large literature on response biases in survey data has demonstrated reliable limits on introspective judgements [5–7,9]. Potential issues with the use of confidence ratings have also been highlighted in recent debates on the extent to which recognition memory ROC functions are compatible with a dual high threshold model positing discrete ‘recognize as old’, ‘recognize as new’ and ‘uncertain’ states, rather than continuously varying evidence assumed by SDT [51–54].

Given that introspective judgements are costly to obtain, largely reflect response fluency (which is usually assumed to be inversely related to response latency), and may be subject to response biases, we assessed to what extent response latencies conveyed similar information to that obtained from introspective judgements. Even though response times have previously been used in several studies to generate ROC functions in perceptual tasks [11,21,24–37], as far as we are aware, the only published application to recognition memory is a study that investigated performance in a range of memory tasks for four participants [23]. This is particularly surprising given that many studies of recognition memory place a strong focus on the shape of ROC functions in an effort to constrain theoretical accounts and given that response times to recognition decisions feature prominently in attempts to understand recognition memory using sequential sampling models; see [55,56] for recent efforts to validate the unequal-variance assumption in recognition memory (which is usually supported by analyses of the shape of ROC functions) with response times in a diffusion model analysis. Furthermore, applied work on eye-witness memory has found response latency to be indicative of identification accuracy [57].

We found that L-ROC functions tended to be closer to the corresponding random ROC functions than C-ROC functions—a common finding among studies using L-ROC functions—but that areas under both types of ROC functions conveyed similar information about relative performance. In all cases of which we are aware, the generation of L-ROC functions depends on the assumption that RT tends to be inversely related to evidence strength. See [38,42] for theoretical work assuming that response time should be related to the signal underlying the classification decision, with the former authors providing a formal derivation of (among other things) how variability in the dependent measure (‘criterion variability’) affects the shape of the resulting ROC function. Even though there is strong evidence for this assumed relationship between response time and evidence strength, there is also evidence for different relationships in some cases [43]. Examination of L-ROC AUCs for individual sessions (not shown here) revealed that in some cases these AUCs fell considerably below 0.5 (i.e. the level indicating no relationship between RTs and memory strength). This suggests that for these cases our assumptions for the calculation of RT strength are not met. Analyses of some of these cases (not reported in detail here) revealed relatively high FA rates and a tendency for faster responses (particularly for incorrect responses). This is evidence for a substantial proportion of fast guesses in these data which would counteract the assumed trend of shorter RTs being associated with stronger evidence. Presumably inferential processes that generate confidence ratings on the basis of response fluency can take into account when a response was guessed quickly which may help explain the absolute differences in the AUCs for ROC functions based on confidence ratings and response latencies. Note also that some sessions contained more targets than lures which may have led participants to preferentially guess ‘old’. Such a response pattern could explain the relatively larger AUCs for ‘old’ confidence responses shown in figure 2*b* (figure 2*c* shows no such advantage for ‘new’ confidence responses). Additionally, analyses using discrete-state models of recognition memory suggest that detect-old states are more common than detect-new states and that detect-old states are associated with more extreme confidence ratings [51,58]. It may well be possible to account for the relatively larger AUCs for ‘old’ confidence responses on the basis of these findings, but this would require further modelling.

It is likely that even the data without obvious violations of the assumptions for the calculation of RT strength are somewhat contaminated by fast guesses. These tend to reduce the area under L-ROC functions unless the calculation of RT strength is amended to identify these cases to either exclude them or to associate them with low levels of evidence. Modelling the relationship between RT and memory strength in a way that allows for variability between participants and sessions might provide a sensible alternative basis for computing L-ROC functions. Recent accounts of confidence ratings in recognition memory and other binary choice tasks have successfully captured accuracy and response time data with sequential sampling models [43,59]. The RTCON model [43] is designed to handle confidence ratings only without provisions for a separate binary choice and is thus, in its current form, not able to account for similarities (or lack thereof) between C- and L-ROC functions. The two-stage dynamic signal detection interrogation model of confidence [59], on the other hand, models both binary classification decisions and subsequent confidence ratings by assuming that accumulating evidence is assessed twice: once with respect to binary classification criteria and then again, at a later point, with respect to confidence criteria. This set-up predicts a correlation between confidence ratings and binary choice RTs such that more confident responses are associated with shorter binary choice RTs. The strength of this relationship depends on the exact parametrization of the model and it is conceivable that this model, or a close variant, could provide the basis for a better way to generate L-ROC functions taking into account individual differences.

ROC functions that were specific to each classification response (figure 3) provide a novel way to assess to what extent a given measure qualifies the evidence underlying each possible response. It is conceivable that some measures are differentially sensitive to different levels of evidence or that evidence associated with one response category is inherently less variable (perhaps because of a threshold process rather than a smooth transition from lower to higher levels of evidence). Indeed for both confidence and latency data, we found that the areas under the ROC functions for ‘new’ responses were considerably smaller than the corresponding areas for ‘old’ responses. This indicates that both confidence ratings and response times, are better able to qualify ‘old’ than ‘new’ responses, but these analyses are unable to determine to what extent this is because of differential sensitivity of these measures to evidence levels associated with the two response classes and/or properties of the underlying evidence distributions. In this context, it is interesting to note that especially for items classified as ‘old’, both confidence ratings and response latencies were considerably better able to qualify the classification decision for recalled relative to unrecalled items. Consistent with the comparison between ‘old’ and ‘new’ responses discussed above, this result indicates that both measures are more effective when operating on higher levels of evidence—a finding that, again, could be owing to properties of these measures and/or the evidence distributions. A common finding in applications of SDT to recognition memory data is that estimates of the variability of memory strengths for targets tend to be larger than those for lures. Our finding of larger AUCs for ‘old’ responses compared with ‘new’ responses is compatible with this finding.^3^ Indeed, the fact that we found this pattern for both confidence ratings and response times supports this result without relying on the assumptions of a particular modelling framework such as SDT or a drift-diffusion model [55,56].

Traditionally, researchers have placed a strong focus on the exact shape of the ROC function in an effort to draw inferences about the underlying cognitive processes. Such inferences depend on a number of strong assumptions about the cognitive processes underlying the classification decision as well as about the mapping between latent states, e.g. ‘memory strength’ and the variables used to measure them (e.g. confidence ratings). We already alluded to alternatives to our assumptions regarding the mapping between memory strength and response latency—changes in these assumptions will produce corresponding changes in the shapes of the resulting ROC functions that are not related to properties of the cognitive processes underlying the classification decision. Despite the higher face validity for the assumptions regarding the mapping between memory strength and introspective ratings (e.g. a higher confidence rating for an ‘old’ response corresponds to a stronger memory), it is important to remember that this mapping can also be distorted [5–7,9,16], which likewise limits the diagnosticity of the corresponding ROC function with respect to the cognitive processes underlying the classification decision. Furthermore, different dependent measures are likely to respond differently to some experimental manipulations—speed stress, for example, is likely to affect response latencies more than confidence ratings, whereas instructions to distribute responses equally among the response alternatives may produce a more uniform distribution of confidence ratings at the expense of a reduced correspondence between those ratings and the latent state they are meant to assess. Because the details of the mapping between a given latent state and the dependent variable used to assess it crucially affect the shape of the corresponding ROC function, attempts to draw conclusions about the cognitive processes underlying the classification decision from the shape of the ROC function need to be justified for the particular context from which the ROC function was derived. Indeed, even the assumptions that are not specific to the particular dependent variable used to generate the ROC function have been criticized as ‘difficult-to-justify and untestable’ [54] and some of these assumptions have been shown to not hold up to scrutiny [8,43,60,61]. Our approach mostly sidesteps these issues by focusing on the area under the ROC functions to qualify the classification decision. As described above, violations of the assumptions regarding the mapping between the latent state and the dependent variable used to measure it will affect the corresponding ROC functions (and thus the respective AUCs), but unless the extent of this violation co-varies with other variables under consideration, this should only affect the absolute value of the AUCs and not the relative pattern of AUCs across those variables.

A common purpose of SDT analyses is to assess to what extent a given experimental manipulation affects the ability to discriminate between the stimulus classes ‘sensitivity’ and/or the preference for a given response option ‘response bias’. The assessment of response biases requires a model of the decision process, but the current method allows the direct assessment of discrimination performance without the need to subscribe to the specific assumptions of SDT. In this context, it is important to note that differences in average response latency across the two response classes (perhaps owing to a response bias favouring one response class over the other) do not affect the resulting L-ROC functions. This is owing to the fact that during the construction of the ROC function, the dependent variable is sorted separately for each response class such that a latency corresponding to strong evidence when it refers to one response option may correspond to weak evidence when it refers to the other response option (owing to a larger proportion of faster responses). Similarly, in cases where different ranges of nominal confidence levels are used for different response classes, these would usually be interpreted such that the largest values within each response class correspond to the most extreme evidence states, without consideration for consistency of the nominal confidence levels across response options.

Several of our analyses correlated different subsets of the data (partitions based on ‘old’ versus ‘new’ responses and/or recalled versus unrecalled targets) for measures based on both confidence ratings and response latency (cf. figures 4, 6 and 7). All of these correlations were strikingly similar for measures based on confidence ratings and response latency, in both cases showing strong correlations between AUCs for recalled and unrecalled items (figure 6), especially for items classified as ‘old’ (figure 7), as well as between AUCs for ‘old’ and ‘new’ responses (figure 4). This suggests that the internal structure of the data based on both dependent measures was quite similar, despite the absolute differences in the resulting AUCs—a result that supports our earlier assertion that relative differences in performance are captured similarly well by measures based on confidence ratings and response latencies.

## 5. Conclusion

The method we used to generate ROC functions can be (and has been) applied to other classification tasks and to other dependent variables besides confidence ratings and response times. It is likely that measures not based on confidence ratings or response latencies can provide similar or better insights into the evidence underlying classification responses and the current framework allows for the quantitative assessment of such (potential) indices of cognitive states (relative to a random baseline and/or to another index). ROC functions undeniably play an important role in the assessment of choice behaviour—a role that generalizes well beyond tests of theories of recognition memory. In some cases, collection of confidence ratings can be impractical and the collection of binary choices across multiple conditions is not always a viable alternative. A strength of the current approach is that it generalizes over a wide range of potential tasks and dependent measures to assess the evidence underlying classification responses. We have shown that L-ROC functions derived from RTs to binary choice responses in a recognition memory task provide similar information about discriminability to that obtained from confidence ratings. In this process, we have presented a novel method to separately assess the information revealed by a dependent measure with respect to each response class. Importantly, this way of evaluating evidence for classification decisions, either across all responses or separately for each response class, can provide detailed insights into how experimental manipulations affect cognitive processing without relying on assumptions regarding the distribution of evidence or the nature of the decision process. The ability to use incidental measures (such as RT) for this purpose provides a convenient alternative to traditional ways of computing ROC functions that is not subject to the limits of introspection and that can even be applied to experiments ‘retrospectively’ as long as RT data (or data from another dependent variable that is thought to reflect the cognitive state on which a classification decision is based) is available.

## Supplementary Material

WeidemannKahana2016_data.hdf5

## Supplementary Material

data file for ANOVA main_analyses.py

## Supplementary Material

main data file recunrecANOVA_sess.tsv

## Supplementary Material

main analysis script recunrecANOVA.R

## Supplementary Material

script to calculate ANOVA

## Footnotes

1 In certain situations, the relationship between response times and signal strength is better described by a speed-accuracy trade-off and conditional accuracy functions are sometimes used to characterize this relationship, especially in cases where response speed is not manipulated (ch. 6 of [22] discusses these cases and analyses).

2 The interaction between recalled status and dependent measure also reached conventional levels of statistical significance, but that effect was of negligible size: $\eta_{p}^{2}=0.06$.

3 The exact relationship between the relative variability of memory strengths for targets and the AUCs for ROC functions corresponding to ‘old’ versus ‘new’ responses depends on a number of assumptions, including the placement of the classification criterion, but simulations confirmed that within the framework of a simple SDT model, larger target variability coincided with larger AUCs for ‘old’ responses.

## Data accessibility

The dataset used in this article as well as associated analysis scripts (for Python 2.7 and R) are available as electronic supplementary material and also on both authors’ websites (http://cogsci.info and http://memory.psych.upenn.edu/). The data are provided in the open HDF5 format. A free C++ library to interact with HDF5 files is available from the HDF group (https://www.hdfgroup.org) and free wrappers for this library are available for other programming languages (e.g. h5py for Python http://www.h5py.org/ or the h5 package for R; Matlab provides the h5read command for this purpose). The HDF Group also provides a range of free tools to directly interact with HDF5 files such as HDFView (https://www.hdfgroup.org/products/java/hdfview/).

## Appendix A. Procedures for calculating receiver operating characteristic functions

Table 1 contains data from a fictional recognition memory experiment to illustrate the construction of the different types of ROC functions used in this article. Classification responses are partitioned on the basis of a measure (such as confidence ratings or response times) that qualifies this decision. This partitioning is based on quantiles of this variable. The partitioning in table 1 is compatible with binary classification responses followed by a confidence rating with five levels (a rating of ‘5’ would correspond to 100th percentile of possible ratings, a rating of ‘4’ to the 80th percentile of possible ratings, etc.). For continuous variables such as response times, the number of possible values is infinite, but the ROC function is fully described as long as the number of percentiles is at least as large as the number of unique values that were measured (i.e. no more than the number of trials).

**Table 1.** Number (first set of rows) and proportions (bottom two sets of rows) of responses in a fictional recognition memory experiment broken down by percentiles of a measure (such as confidence ratings or response times) qualifying the classification response. (The sums (*Σ*) of ‘new’ and ‘old’ responses are indicated for the different stimulus classes along with the overall number of targets and lures. The first set of rows (labelled ‘RAW’) contains counts of the number of responses. The middle set of rows (labelled ‘ALL’) contains the same data normalized by the respective total numbers of target and lure trials. The final set of rows (labelled ‘REC’) contains the same data normalized by the respective numbers of target and lure trials within each recognition response.)

		‘new’						‘old’						
percentile		100	80	60	40	20	*Σ*_‘new’_	20	40	60	80	100	*Σ*_‘old’_	*Σ*
RAW	lure	24	20	14	12	10	80	8	6	3	2	1	20	100
	target	1	2	3	5	4	15	9	12	15	20	29	85	100
ALL	lure	0.24	0.20	0.14	0.12	0.10	0.80	0.08	0.06	0.03	0.02	0.01	0.20	1
	larget	0.01	0.02	0.03	0.05	0.04	0.15	0.09	0.12	0.15	0.20	0.29	0.85	1
REC	lure	0.300	0.250	0.175	0.150	0.125	1	0.400	0.300	0.150	0.100	0.050	1	2
	target	0.067	0.133	0.200	0.333	0.267	1	0.106	0.141	0.176	0.235	0.341	1	2

The raw data in the top set of rows in table 1 is normalized in two different ways in the bottom two sets of rows. The normalization in the middle row (labelled ‘ALL’) corresponds to the histograms shown in figure 1*a* and the normalization in the bottom row (labelled ‘REC’) corresponds to the histograms shown in figure 3*a* (albeit for the fictional data from table 1 and not for the actual data presented in the article).

Table 2 lists the values for the standard ROC function based on both classification responses. It can be derived by cumulatively adding the respective values from table 1 starting from the strongest evidence in favour of the standard response class (100th percentile ‘old’ responses) to the weakest evidence in favour of the standard response class (100th percentile ‘new’ responses). The resulting ROC function is plotted in figure 8*a*.

**Table 2.** Cumulative hit and false alarm (FA) rates for different strength criteria inferred from the fictional data in table 1. (The top set of rows (labelled ‘RAW’) contains the raw frequencies and the bottom set of rows (labelled ‘ALL’) contains the same data normalized by the total number of targets (for hits) and lures (for FAs).)

		strength criterion										
		liberal			........................					conservative		
RAW	FA	100	76	56	42	30	20	12	6	3	1	0
	hit	100	99	97	94	89	85	76	64	49	29	0
ALL	FA	1	0.76	0.56	0.42	0.30	0.20	0.12	0.06	0.03	0.01	0
	hit	1	0.99	0.97	0.94	0.89	0.85	0.76	0.64	0.49	0.29	0

Tables 3 and 4 show the values for the ROC functions conditioned on ‘new’ and ‘old’ responses, respectively. These can be derived by cumulatively adding the respective values from the bottom set of rows in table 1 within each classification response starting from the strongest evidence in favour of the standard response class (100th percentile ‘old’ responses or 20th percentile ‘new’ responses) to the weakest evidence in favour of the standard response class (20th percentile ‘old’ responses or 100th percentile ‘new’ responses). The resulting ROC functions are plotted in figure 8*b*. The area under the ROC functions can be calculated by adding the areas defined by each pair of points and their projections onto the ordinate.

**Table 3.** Cumulative hit and false alarm (FA) rates for different strength criteria inferred from the fictional ‘new’ responses in table 1. (The top set of rows (labelled ‘RAW’) contains the raw frequencies and the bottom set of rows (labelled “new”) contains the same data normalized by the total number of targets (for hits) and lures (for FAs) for which a ‘new’ response was made.)

		‘new’ strength criterion					
		liberal		......	conservative		
RAW	FA	80	56	36	22	10	0
	hit	15	14	12	9	4	0
‘new’	FA	1	0.700	0.450	0.275	0.125	0
	hit	1	0.930	0.800	0.600	0.267	0

**Table 4.** Cumulative hit and false alarm (FA) rates for different strength criteria inferred from the fictional ‘old’ responses in table 1. (The top set of rows (labelled ‘RAW’) contains the raw frequencies and the bottom set of rows (labelled “old”) contains the same data normalized by the total number of targets (for hits) and lures (for FAs) for which an ‘old’ response was made.)

		‘old’ strength criterion					
		liberal		......	conservative		
RAW	FA	20	12	6	3	1	0
	hit	85	76	64	49	29	0
‘old’	FA	1	0.600	0.300	0.150	0.050	0
	hit	1	0.894	0.753	0.576	0.341	0
